# DNMT3B overexpression downregulates genes with CpG islands, common motifs, and transcription factor binding sites that interact with DNMT3B

**DOI:** 10.1038/s41598-022-24186-6

**Published:** 2022-12-02

**Authors:** Jaqueline Loaeza-Loaeza, Angel Josué Cerecedo-Castillo, Hugo Alberto Rodríguez-Ruiz, Yaneth Castro-Coronel, Oscar Del Moral-Hernández, Félix Recillas-Targa, Daniel Hernández-Sotelo

**Affiliations:** 1 Laboratorio de Epigenética del Cáncer, Facultad de Ciencias Químico Biológicas, Universidad Autónoma de Guerrero, Av. Lázaro Cárdenas S/N Col. Haciendita, 39070 Chilpancingo, Guerrero Mexico; 2 Departamento de Genética Molecular, Instituto de Fisiología Celular, Universidad Nacional Autónoma de México, 04510 Ciudad de México, Mexico; 3 Laboratorio de Biomedicina Molecular, Facultad de Ciencias Químico Biológicas, Universidad Autónoma de Guerrero, Av. Lázaro Cárdenas S/N Col. Haciendita, 39070 Chilpancingo, Guerrero Mexico; 4 Laboratorio de Citopatología e Inmunohistoquímica, Facultad de Ciencias Químico Biológicas, Universidad Autónoma de Guerrero, Av. Lázaro Cárdenas S/N Col. Haciendita, 39070 Chilpancingo, Guerrero Mexico; 5 Laboratorio de Virus y Cáncer, Facultad de Ciencias Químico Biológicas, Universidad Autónoma de Guerrero, Av. Lázaro Cárdenas S/N Col. Haciendita, 39070 Chilpancingo, Guerrero Mexico

**Keywords:** Cancer, Genetics, Molecular biology

## Abstract

DNA methylation is a key epigenetic modification to regulate gene expression in mammalian cells. Abnormal DNA methylation in gene promoters is common across human cancer types. DNMT3B is the main de novo methyltransferase enhanced in several primary tumors. How de novo methylation is established in genes related to cancer is poorly understood. CpG islands (CGIs), common sequences, and transcription factors (TFs) that interact with DNMT3B have been associated with abnormal de novo methylation. We initially identified *cis* elements associated with DNA methylation to investigate the contribution of DNMT3B overexpression to the deregulation of its possible target genes in an epithelial cell model. In a set of downregulated genes (n = 146) from HaCaT cells with DNMT3B overexpression, we found CGI, common sequences, and TFs Binding Sites that interact with DNMT3B (we called them P-down-3B). PPL1, VAV3, IRF1, and BRAF are P-down-3B genes that are downregulated and increased their methylation in DNMT3B presence. Together these findings suggest that methylated promoters aberrantly have some *cis* elements that could conduce de novo methylation by DNMT3B.

## Introduction

DNA methylation in a CpG context is a key epigenetic process that regulated gene expression. DNA methylation consists of a chemical addition of a methyl group on the fifth carbon in cytosines. In a structural sense in the genome, regions with extensive CpG contain, called CGIs exist. The CGIs have an average size of 1 kb, and a CpG content > 60% and they are prevalently found in promoter regions^1^. Cytosine methylation increases the contained information in the genetic sequence, in this sense, the same sequence might be read differentially and participate in the on/off switch from specific genes^2^. CpGs methylation in humans is deposited by DNA methyltransferases (DNMTs) enzymes^3^. DNMT1 is a maintenance DNMT and establishes DNA methylation in hemimethylated substrates, mainly, during DNA replication, and is abundant in somatic cells. DNMT3A and DNMT3B are de novo DNMTs that establish DNA methylation in hemimethylated and unmethylated substrates and their levels of expression are higher in embryonic cells^4–6^. DNA methylation has been proposed to regulate gene expression during development and maintain fate cells, but deregulation of any of the 3 catalytically active methyltransferases in embryonic or somatic cells, leads to aberrant gene expression and results in human diseases^7,8^. De novo methylation is predominantly established by DNMT3A and DNMT3B, although both share function and similar targets, there are specific target regions and genes for each of them^9^, different functional mechanisms^10,11^, differential functional results in the same context^12^ and, there are different expression profiles in cancer for each of DNMTs^13^. In several solid tumors, DNMT3B is upregulated, correlates with the epigenetic inactivation of tumor suppressor genes, and increases the number and volume of tumors^14^. The human genome is segmented in biological *cis* regulatory elements (promoters, enhancers, CGIs, TFBS, and others), which are a key class of regulatory noncoding DNA sequences, which act to regulate the transcription of a neighboring gene^15^. The direct regulation of DNMT3B over their target genes has been sparsely tested but exist reports of *cis* elements related to de novo methylation as CGIs, TFs, and common motifs of methylated promoters. These elements have been found in genes that are downregulated by DNMT3B.

The CpG content on the human promoters has a critical role in DNA methylation in somatic cells. Commonly, promoters with high content of CpG (HCP) are sparsely methylated and are expressed. Promoters with intermediate content of CpGs are dynamically methylated, and their transcriptional state is regulated via methylation depending on the tissue, state of differentiation, and cell cycle. Promoters with low content of CpGs (LCP) are methylated and their target genes are repressed transcriptionally^16,17^. The CpG context in cancer becomes relevant for the establishment of abnormal methylation patterns by DNMTs, there is DNA methylation gain in CpG rich regions or CGIs^18^. When the DNMT3B expression is increased, also is enhanced de novo methylation^19^, DNMT3B preferential binding, and its enzymatic activity in CGIs^20,21^.

De novo methylation has been related to the information contained in the own *cis* sequences and has been reported that sequences adjacent to CpG are involved in the affinity of the human DNMTs for de novo methylation^22–24^. DNA methylation deposited by DNMT3B has been observed on the CANAGCTG sequence (N, any nucleotide)^25^. Analysis de novo motifs elucidation for the methylated regions by DNMT3B showed an enrichment of the thymine residue in position − 1 and guanine residue in position + 1 in the motif found for these regions, NTCpGGN^26^. The guanine presence in the + 1 of CpG preferred by DNMT3B was recently confirmed by biochemical assays where specific amino acids (Asn779 and Lys777) of the DNMT3B catalytic domain are involved^27^. The CGIs are considered biologically active motifs and platforms for the recruitment of chromatin-modifying proteins that facilitate transcription regulation. In the CGIs, we found many TFBS, and some of them are transcription repressor factors. There are several TFs that interact with DNMTs and regulate target genes^28–31^. RASSF1A is a gene that can be regulated by DNMT3B via DNA methylation in cancer. Myc is a TF associated with oncogenic functions and has been described as favoring the recruitment of DNMT3B to the RASSF1A promoter, which results in its epigenetic silencing and improves its function as a tumor suppressor^32^. Also, in prostate cancer, REX1 overexpression recruits DNMT3B and downregulates RASSF1A by promoter methylation^33^.

There is evidence for the overexpression of DNMT3B and its correlation with the epigenetic inactivation of tumor suppressor genes^34^. Several key genes for cell normality have been found methylated, and interestingly, DNMT3B in the mouse colon initiates de novo methylation of genes that are like genes that become methylated in human colon cancer. In the previous work, they suggest that a possible explanation for this similarity is that aberrant methylation in cancer can be attributed to the targeting of specific sequences rather than random methylation^35^. Additionally, in cancer, has been found that DNMT3B overexpression contributes to a hypermethylator phenotype^36^.

To gain knowledge about DNMT3B overexpression and downregulated genes, we focus on common elements, function, expression, and methylation statuses in a gene set. We identified *cis* elements in this gene set downregulated by DNMT3B overexpression in HaCaT cells. We select specifically four genes with *cis* elements and found decreased expression and promoter methylated in PPL1, VAV3, IRF1, and BRAF. Taken together, our data suggest that an abnormal increase of DNMT3B conduces to aberrant de novo methylation and this event can be influenced by *cis* elements found in possible DNMT3B target genes.

## Results

### Genes downregulated by DNMT3B overexpression have common cis elements

**Figure 1:**
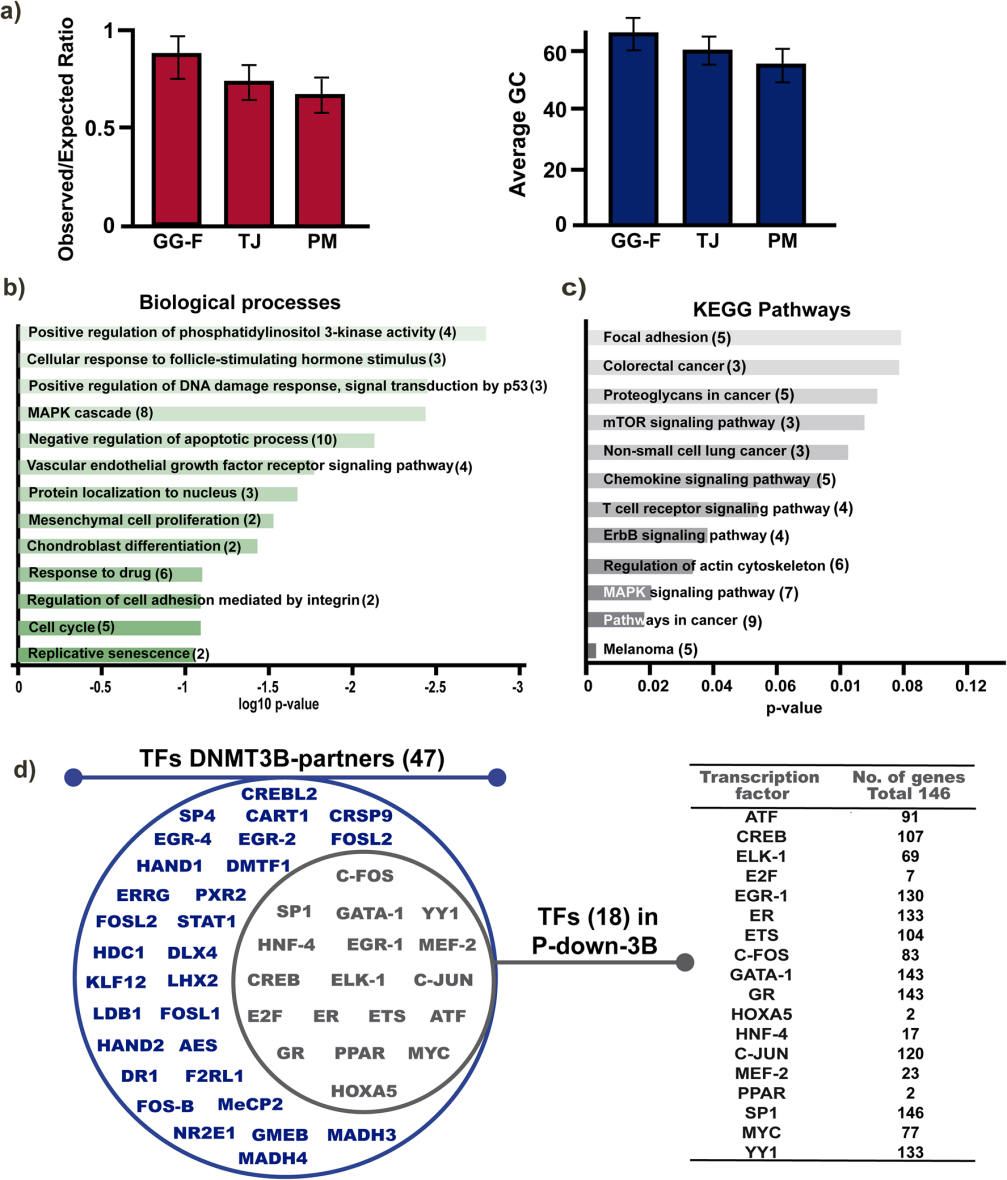
Overexpression of DNMT3B decreases expression of genes related to cancer and with *cis* elements presents in methylated promoters. (**a**) Observed/expected ratio and CpG average from CpG islands found in P_Down_CGI identified with three algorithms: GG-F, Gardiner-Garden and Frommer algorithm; TJ, Takai-Jones algorithm; PM, Ponger-Mouchiroud algorithm. (**b**) GO biological process complete and (**c**) KEGG pathways significantly enrichment of 136 genes with decreased expression (Z score 2.0 to 6.8) by DNMT3B overexpression and CGI on its regulatory region (P-down-3B). (**d**) Venn diagrams (left) show all TFs found that interact directly with DNMT3B and the TFs fraction that have binding sites and enrichment on P-down-3B genes. TFs have distinct frequency distribution (right) in the P-down-3B genes set.

Our workgroup previously generated a DNMT3B overexpression model in HaCaT cells. Through an expression microarray, we found 252 downregulated genes by DNMT3B overexpression with a z-score of 2 to 6.8^37^. In possible DNMT3B targets genes with decreased expression, our first goal was the identification of *cis* elements that can influence de novo methylation. Due to the repressor function of DNMT3B and the CpG-context influence on de novo methylation, we first identified genes with CGI as a possible element involved in DNMT3B targeting downregulated genes. Through three algorithms, we found 146/252 genes with CpG islands in their promoter region. These CGIs have an observed/expected ratio > 0.6 and a CpG average greater than 50% (Fig. 1a).

DNMT3B overexpression led to abnormal methylation in genes with key roles in the biological normal process^38,39^. To know the processes which are involved in the downregulated genes with CGI, we made a gene ontology (GO) and Panther pathway analysis. According to GO, the genes participate in the cell cycle, senescence, MAPK cascade, PI3K regulation, apoptosis, and others (Fig. 1b). In KEGG analysis, the downregulated genes were found in cancer pathways, MAPK and ErbB signaling, melanoma, colorectal and non-small cell lung cancer, etcetera (Fig. 1c).

In addition to the CGIs, has been described that in some promoters, the recruitment of DNMTs might be directed by interaction with TFs^32^. We question if downregulated genes (with CGI) might have binding sites for TFs that interact directly with DNMT3B. We found in the literature 47 TFs that shape complex with DNMT3B (Fig. 1d, left)^40^. Bioinformatic analysis was performed with ALIBABA 2.0, Expasy (EPD), and genome browser data for the binding sites identification of TFs on the regulatory sequences of DNMT3B downregulated genes (Fig. 1d, left). We found 18/47 TFBSs previously reported with DNMT3B interaction (TFs DNMT3B-partners) in the promoters of analyzed genes. We found binding sites for TF as SP1, EGR-1, ER, GATA-1, GR, c-JUN y YY1, in 120 to 146 selected genes. Binding sites for ATF, CREB, ELK1, c-FOS y MYC, that bond to 107 to 77/146 genes, and some TFs with scarce binding sites; E2F, HOXA5, HNF4, MEF2 y PPAR that are found in 2 to 23 genes (Fig. 1d, right). The downregulated genes by DNMT3B overexpression with CpG islands and TFBS were named P-down-3B genes.

**Figure 2:**
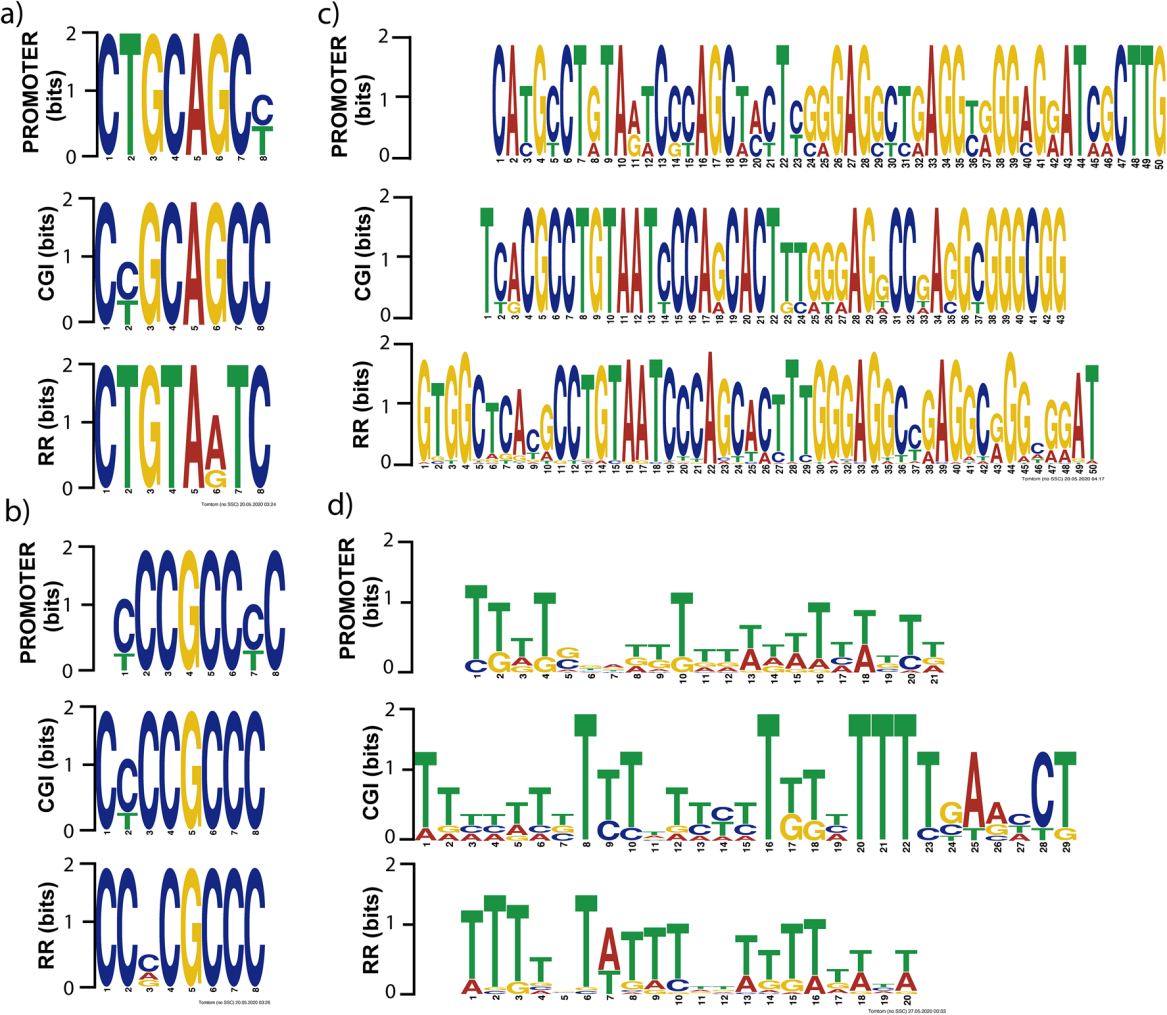
DNMT3B downregulated genes have common motifs in their functional regulatory regions. Common sequences found with MEME Suite in P-down-3B genes. (**a**) and (**b**) short and ungapped motifs determinate with DREME in RR, CGI, and PROMOTER. (**c**) The top De Novo motif found with MEME. (**d**) The top motif discovered with MEME ChIP (Human-Methylcytosine, Yan 2017) in the three functional regions from P-down-3B.

Hitherto, possible binding sequences have been reported for DNMT3A or DNMT3B in particular contexts. These sequences are found in methylated promoters in presence of DNMT3A or DNMT3B^25^. We believe that might there are sequences in the downregulated genes that reflect preference by DNMT3B. Therefore, we perform motif analysis with MEME Suite to identify common motifs in P-down-3B genes. We search motifs in three functional regions from P-down-3B genes; the regulatory region −2000 to 1000 (RR), the CpG island (CGI), and de closely promoter (−400 to 100, (PROMOTER)). The short and ungapped motif elicitation showed several common motifs between the 3 functional regions. We show two short representative motifs highly like each other (Fig. 2a,b). The discovery of novel and ungapped motifs showed a large common motif in the RR, CGI, and Promoter (Fig. 2c). The comprehensive motif analysis in Human 5-methylcytosine for transcription factor binding to methylated DNA show a similar motif in P-down-3B genes (Fig. 2d). These possible preferentially sequences for DNMT3B were analyzed in the146 P-down-3B genes and were found in at least 80% of them.

### DNMT3B overexpression is common in cancer patients from TCGA data sets

**Figure 3:**
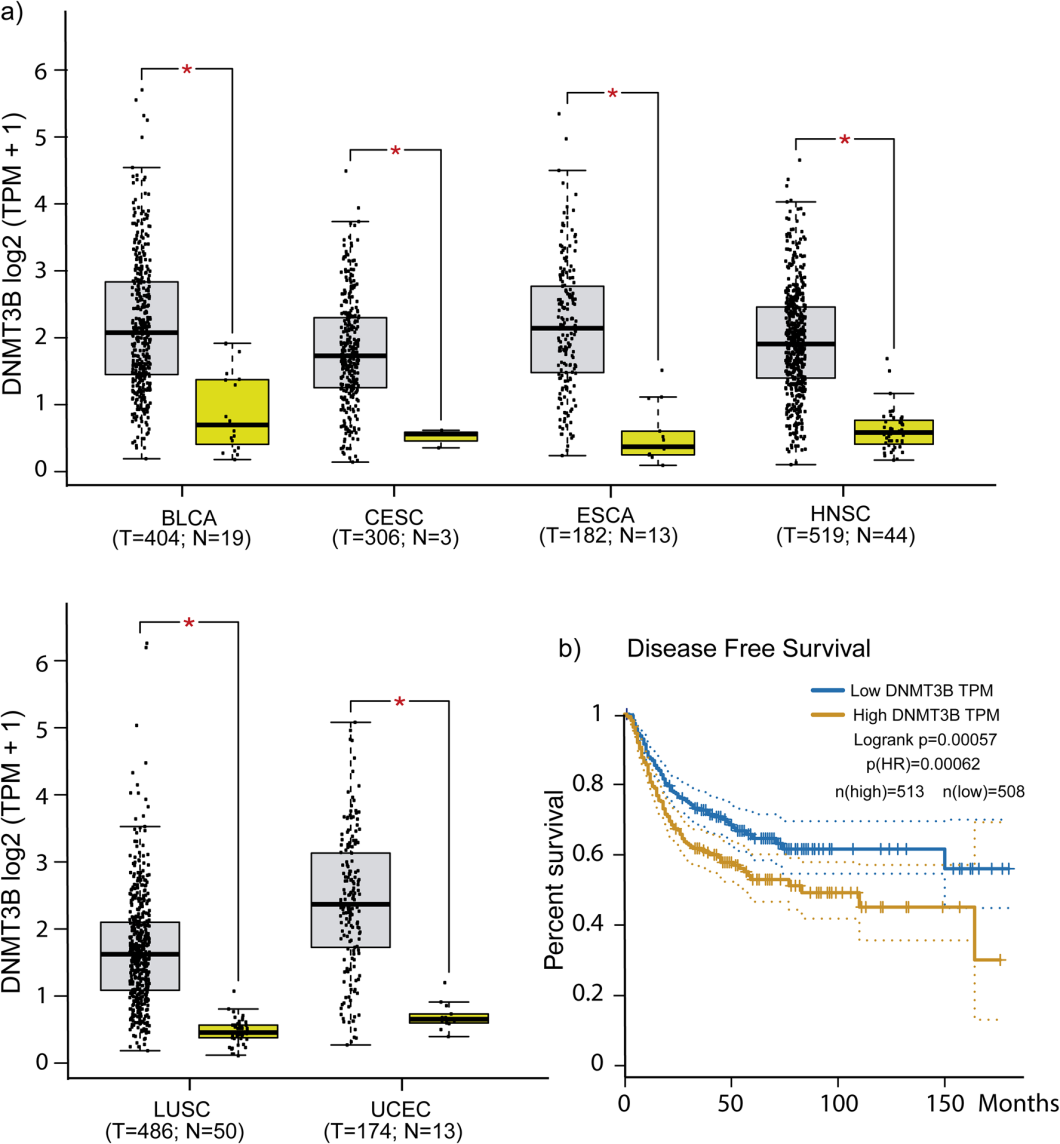
DNMT3B is overexpressed in tumors of patients from TCGA data sets and is correlated with poor prognosis. (**a**) Comparison of the DNMT3B expression in six epithelial carcinomas respect to normal tissue using data retrieved from TCGA. (**b**) Kaplan–Meier curves of disease-free survival for six solid tumors (**a**) with low versus high expression of DNMT3B. BLCA, Bladder Urothelial Carcinoma; CESC, Cervical Squamous Cell Carcinoma and Endocervical Adenocarcinoma; ESCA, Esophageal Carcinoma; HNSC, Head and Neck Squamous Carcinoma; LUSC, Lung Squamous Cell Carcinoma; UCEC, Uterine Corpus Endometrial Carcinoma; T, Tumor; N, Normal tissue.

Next, we examined whether DNMT3B expression is increased in cancer patients. We interrogate the DNMT3B levels and disease-free survival in cancer patients in TCGA data sets. DNMT3B is overexpressed significantly in six representative epithelial human solids tumors (BLCA, CESC, ESCA, HNSC, LUSC, and UCEC) concerning normal tissue (Fig. 3a). The survival analysis results from Kaplan–Meier plots depicted the association between DNMT3B levels expression and cancer prognosis; high DNMT3B expression in representative tumors is significantly associated with poor prognosis (Fig. 3b). The same six human tumors were interrogated for DNMT3A levels expression. We found that no there are significant changes in expression in 4/6 cancer samples concerning normal tissue and no significant association was found for the prognosis with respect to DNMT3A expression levels (Supplementary File 2, Supplementary Fig. 1).

### PPL1, VAV3, BRAF, and IRF1 have *cis* elements, gain methylation, and decrease their expression by DNMT3B overexpression

**Figure 4:**
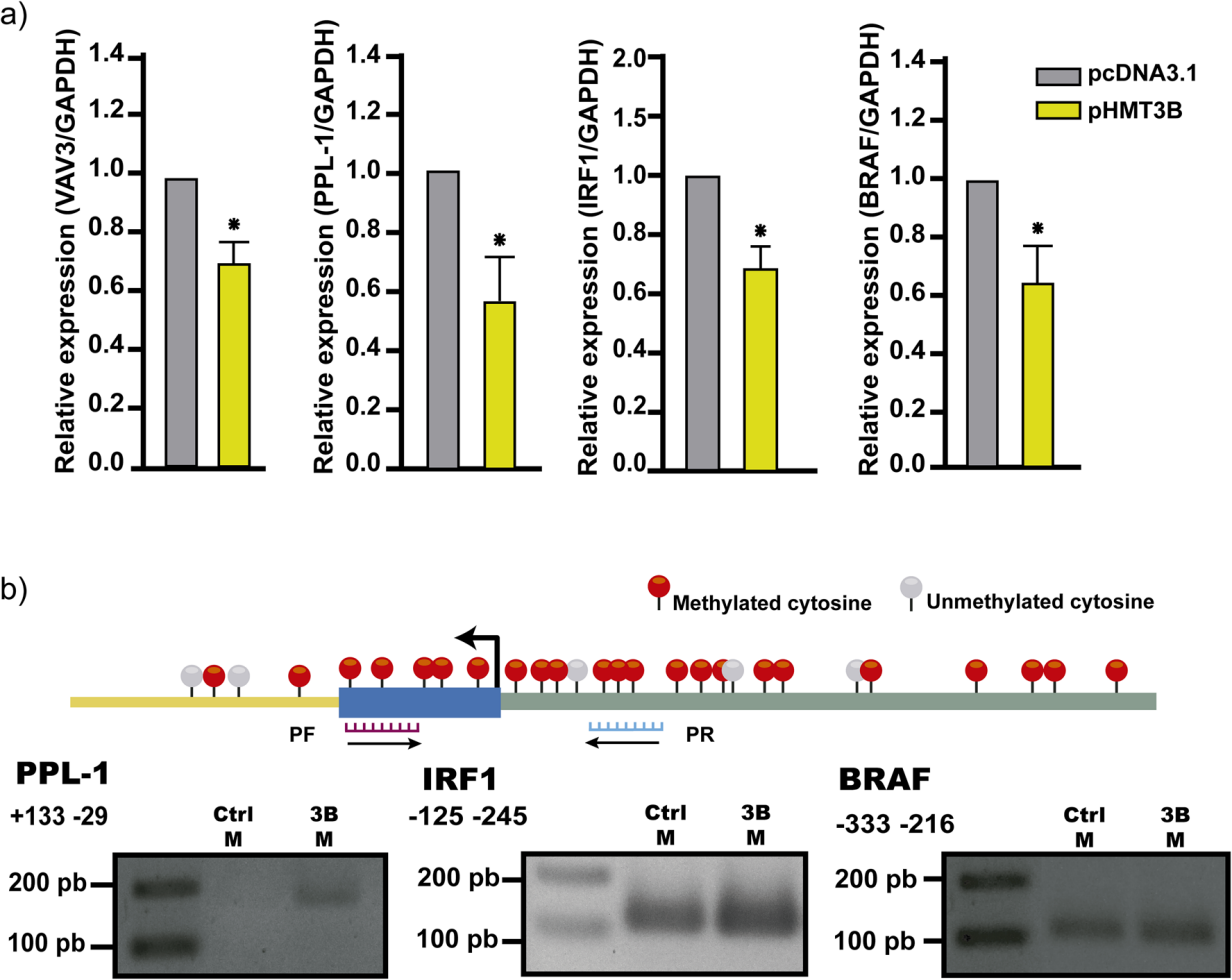
DNMT3B overexpression results in decreased expression and gain methylation of target genes. (**a**) Significative expression changes for VAV3, PPL1, IRF1, and BRAF (left to right) are shown in HaCaT cells with DNMT3B overexpression (pHMT3B) with respect control HaCaT cells (pcDNA3.1). *p > 0.05 of three biological replicas by triplicate. (**b**) Upper, schematic representation of an analyzed region by Methylation Specific PCR with primer for methylated Cytosines. The bottom showed gain methylation in promoter for PPL1, IRF1, and BRAF (left to right) in HaCaT cells with DNMT3B overexpression.

**Figure 5:**
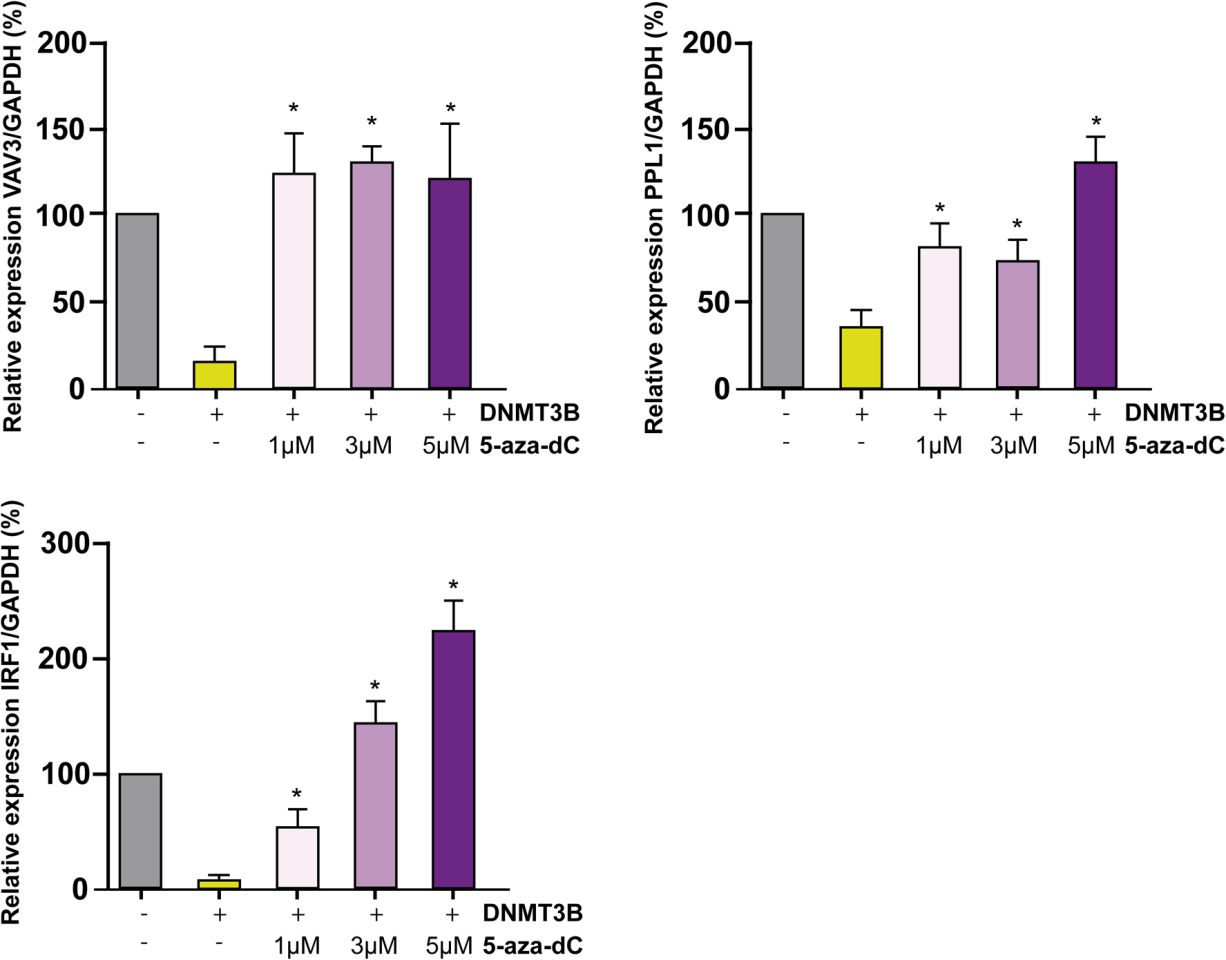
5-aza-dC treatment counteracts the downregulation of possible DNMT3B target genes. The levels of expression of VAV3, PPL1, and IRF1 were evaluated under the following conditions: HaCaT control cells, HaCaT with DNMT3B overexpression, and HaCaT with DNMT3B overexpression treated with 1 μM, 3 μM and 5 μM of 5-aza-dC as is indicated in each graph. The results showed three biological replicates by triplicate.

We next examined some key downregulated genes selected by *cis* elements (Supplementary File 2, Supplementary Fig. 2) and their function in cancer-related processes. We screen the specific transcriptional and methylation state for PPL1, VAV3, BRAF, and IRF1. We wanted to know if these genes decrease their expression and if they gain methylation in HaCaT cells with DNMT3B overexpression. Then, we analyzed the expression of VAV3, PPL1, BRAF, and IRF1 by RT-qPCR and found a decreased expression in the mRNA of these genes (Fig. 4a). The expression of these genes also was decreased in HaCaT cells with endogenous activation and stable expression of DNMT3B (Supplementary File 2, Supplementary Fig. 3). Consequently, we investigate whether the decreased expression in PPL1, BRAF, and IRF1 was due to the gain of methylation in its promoter. We found that in HaCaT cells with DNMT3B overexpression, PPL1, BRAF, and IRF1 gain methylation concerning control HaCaT cells (Fig. 4b). For VAV3, a gain of methylation was previously identified in 2 regions of the VAV3 promoter determined by bisulfite modification and sequencing^37^. To complement the methylation assays, we next conducted experiments with 5-aza-2´-deoxycytidine (5-aza-dC) in HaCaT cells with DNMT3B overexpression. The decreased expression of VAV3, PPL1 e IRF1 by DNMT3B overexpression was restored by the treatment with 1 µM, 3 µM, and 5 µM of 5-aza-dC (Fig. 5).

### DNMT3B overexpression increase cell migration in HaCaT cells

**Figure 6:**
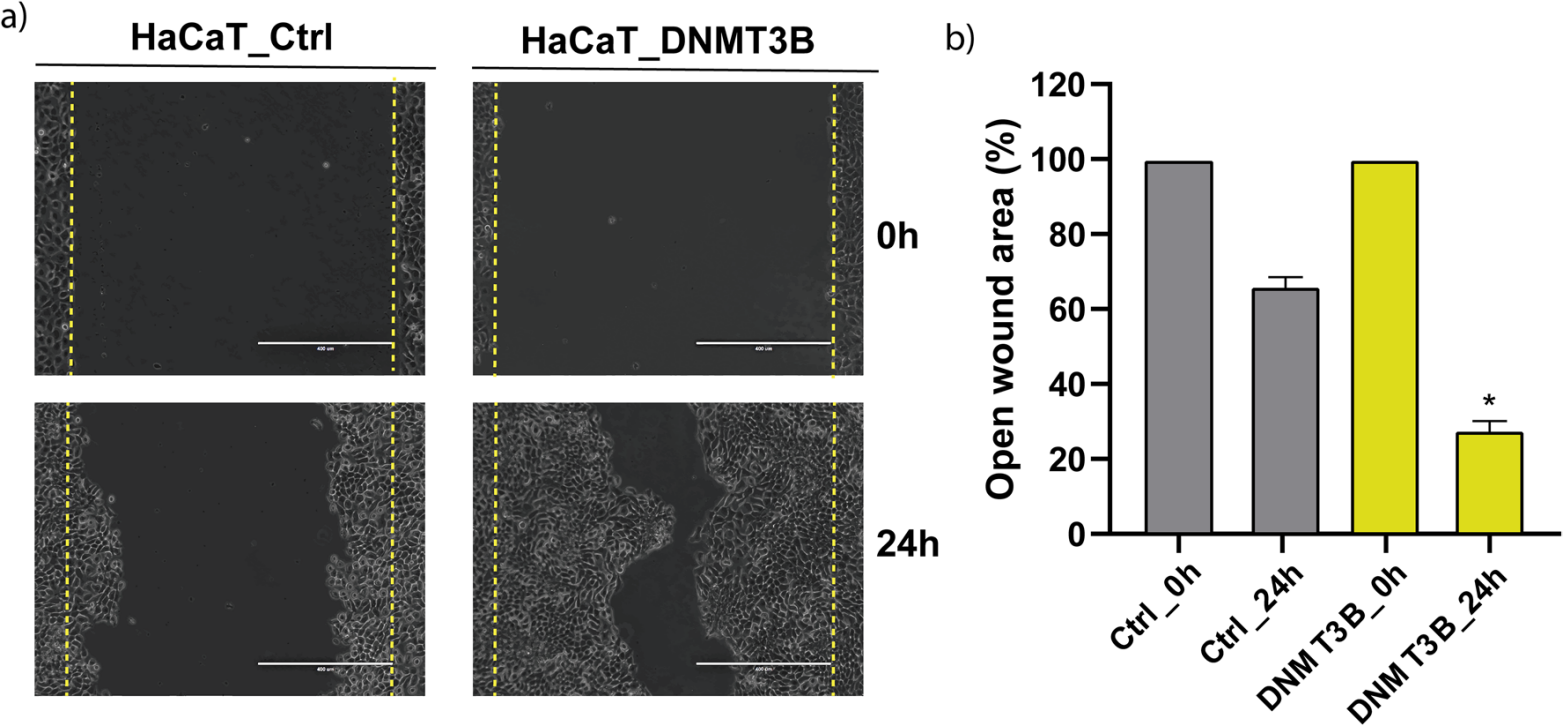
DNMT3B overexpression increased the cell migration. (**a**) Cell migration evaluated by wound closure assays at 0 h and 24 h in control HaCaT and HaCaT with DNMT3B overexpression. (**b**) Quantification of the open area of the wound presented in percentage in each condition. The image is representative of three independent experiments in triplicate.

To highlight the general findings associated with the DNMT3B overexpression that involves several genes and key pathways, we select a common biological process de-regulated in cancer, cell migration. We performed a wound closure assay in control and with DNMT3B overexpression HaCaT cells. We observe a significant increase in cell migration at 24 h with the DNMT3B overexpression concerning control HaCaT cells (Fig. 6a). The quantification of the wound closure showed that DNMT3B overexpression result in a close area of 78% with respect to 35% in control cells (Fig. 6b).

## Discussion

Genes aberrantly silenced by DNMTs overexpression have a negative impact on cell normality. Abnormal methylation patterns in key genes lead to carcinogenic programs in somatic cells^41^. DNMT3B is a key de novo DNMT and its binding at the genome is closely linked to methylation deposition in several functional regions, for example, enhancers^12^, bodies of transcribed genes^20^, intragenic regions^42^, and promoters^43–49^ with different functional consequence. The promoter’s methylation in CGIs is related to gene transcriptional silencing whereas that intragenic methylation is related to gene activation and influences gene isoform mRNA length^50–52^. DNMT3B targets genes in the cancer context and have been described to contain some *cis* elements associated with DNA methylation. In the genomic structural sense, we investigate common *cis* elements in downregulated genes by DNMT3B overexpression and in the functional sense, we evaluated the methylation and expression status of four cancer-related genes and the impact of DNMT3B in a key biological process in cancer.

In this work, we identified that DNMT3B downregulated genes with CGIs in their regulatory region (146 genes), TFBS associated with DNMTs recruitment, and common sequences (Figs. 1 and 2). DNMTs family doesn´t have binding sites in the genome but can be guided to target sites through methylation-prone sequences such as CGIs. The information about the CGIs and CpG content has been successfully used as a predictor of DNA methylation levels in gene promoters. Particularly, DNMT3B preferentially binds to highly repeated heterochromatic regions rich in CpG^53–55^. Taking the evidence together, we believe that DNMT3B´s preference for genes with CGIs could be increased in some diseases where DNMT3B is de-regulated, for example during carcinogenesis^56^. CGIs hypermethylation associated with DNMT3B has been found to increase in cancer and consequently, the methylation of genes with CGI is enhanced^19,57,58^.

The CGIs, being adjacent or within promoters, are also binding platforms for TFs. The methylation of binding sites can favor or prevent the TFs recruitment at their target promoters and consequently regulate the transcription rates^59^. Additionally, some oncogenic TFs complex with DNMTs position themselves on target genes and silence them^32,60,61^. In the literature, there are reports of direct interaction between TFs and DNMT3B, which we found in P-down-3B binding sites for 18 TFs (Fig. 1d). In protein–protein interaction, microarrays found several TFs that interact with DNMTs^40^ and demonstrated that are canonical regulators of the expression of their target genes through DNMT3B recruitment and DNA methylation. In HCC cells, the expression of FOXO1 is repressed by the interaction between FOXM1, RB1, and DNMT3B^62^. Another example is the ZEB1 TF that interacts with DNMT3B, and the consequence is Ddr1 promoter hypermethylation^63^. In breast cancer cells, TBX2 recruits to DNMT3B and other repressor proteins around the NDRG1 proximal promoter^64^. The occupancy by DNMT3B in the HCT116 genome is modulated to subset genes targets of ZBTB24, involved in general cellular maintenance, and coordinately maintains DNA methylation within gene bodies^65^.

Commonly, most proteins that interact with DNA are guided by specific binding sequences. In this sense, some proteins of the methylation cascade such as DNMT3A and TET1 have been reported motif binding in studies of interaction DNA–protein^66,67^. In this study, we found common sequences shared between P-down-3B genes (Fig. 2). To retrieve the information contained in the *cis* sequence, the promoter region, the CpG islands, and the extensive regulatory region of the P-down-3B were scanned separately. In agreement with our results, short common motifs have been reported in methylated promoters by DNMTs overexpression^25,26^ and structural-function assays showed the preference of DNMT3B by CpG with a G in + 1 position^27,68^ similar to found on the complementary strand of Fig. 2b motif. We found a large and highly conserved motif in P-down-CGI independently of the analyzed region (Fig. 2c). The motifs shown in Fig. 2a and Fig. 2d have interesting changes between cytosine/thymine and the abundant presence of only thymine residues, respectively. An explanation for this is that the 5mC is propense to deamination to become thymine, causing C-to-T transitions^69^. Also, the CpG loss is frequently in promoters methylated as a consequence of transitions of methylated cytosines to thymine^70^. The *cis* elements, particularly the *cis* sequence of a promoter, contain the information necessary for DNA methylation establishment^71^. Additional experiments are necessary to validate the direct implication of these *cis* elements in the DNA methylation performed by DNMT3B.

DNMT3B is the main de novo methyltransferase overexpressed in different types of cancer and has been described as necessary for tumor development^13,14,72–74^. With a general approach, we explore a little about the frequency of DNMT3B dysregulation in tumors of epithelial origin. In a data set from TCGA, we see that DNMT3B is the main de Novo methyltransferase overexpressed in epithelial carcinomas (6/6) with respect to DNMT3A (2/6) (Fig. 3 and Supplementary File 2, Supplementary Fig. 1). This can be suggesting that abnormal DNA methylation in these carcinomas is a partial result of DNMT3B contribution and high DNMT3B levels are related to poor prognosis (Fig. 3b). The global DNA methylation added by DNMT3B reflects its importance in cancer and is equally important to know specific genes downregulated by DNMT3B methylation. GST with CGIs in its promoters aren´t generally methylated^16^, but there is a change in methylation patterns during carcinogenesis^75^, and the circumstances or elements that determine it are poorly understood. DNMT3B has several target genes in cancer, including oncogenes and tumor suppressors. In leukemias, the absence or decrease of DNMT3B is an event that favors the malign transformation^76,77^ and has target genes such as C-met^78^. Nevertheless, is most frequently reported that DNMT3B overexpression is closely linked to the silencing of tumor suppressor genes in several tumors^38,39,79–81^. Hitherto, don’t have been reported that the DNMT3B binding in promoters results in an increased gene expression if not quite the opposite, hence, we focus on four downregulated genes with *cis* elements and functionally related to cancer, PPL1, IRF1, BRAF, and VAV3. These genes possess common *cis* elements in abnormally methylated promoters (Supplementary File 2, Supplementary Fig. 2). We found that these genes decrease their expression by DNMT3B increasing and gaining methylation in their promoters’ regions (Fig. 4). Additionally, the levels of expression of these genes were rescued with 5-aza-dC treatment (Fig. 5). PPL1 is a scaffold protein involved in desmosome junctions and keratin filaments in differentiated epithelial cells^82^. PPL1 was reported scarcely expressed in advanced esophageal squamous cell carcinoma (ESCC)^83^, and esophageal cancer^84^. Loss expression of PPL1 in ESCC is associated with hypermethylation and PPL1 induction promoted adhesion and delayed cell migration^85^. BRAF is a gene that codifies for a serine/threonine kinase and is commonly mutated in cancer^86^. It has been linked to the active BRAF protein with the silencing of MLH1, which produces instability in microsatellite regions and hypermutable phenotype^87^. The interferon 1 regulatory factor (IRF1) participates in the transcriptional regulation of genes involved in the mediation of antiviral, immunomodulatory, and antiproliferative effects^88^. IRF1 has been identified as a tumor suppressor in breast cancer^89^. VAV3 is a guanine nucleotide exchange factor involved in the regulation of Rho GTPases and various cellular processes. VAV3 gene has been reported methylated in breast cancer and gastric cancer^90,91^. The gain of methylation and consequently loss of expression of these genes could be associated with a deregulation of their canonical process during carcinogenesis. We are aware that finer experiments are necessary to corroborate these possible DNMT3B target genes. The studies with a wide approach on DNMT3B specific targets in human cancer are limited and most of them are made in human stem cells and animal models. We re-analyzed RNA-Seq data from Human Epidermal Stem Cells (EpSC) with DNMT3B depletion^12^. In these data, we found that PPL1 and IRF1 increase their expression in EpSC with si-DNMT3B (Supplementary File 2, Supplementary Fig. 4). In another study made in the H1 hESC line^92^, the DNMT3B peaks are distributed in different regions of the genome (Supplementary File 2, Supplementary Fig. 5a) and the identification of nearest genes to DNMT3B peaks showed that 57/146 genes with CGI from our work were found near from DNMT3B peaks, included IRF1, BRAF, VAV3, and PPL1. On other hand, only found 23/100 genes downregulated without CGI in the genes set with DNMT3B peaks (Supplementary File 2, Supplementary Fig. 5b). In these peaks, we found 6/18 TFs (Supplementary File 2, Supplementary Fig. 5c) identified on the regulatory sequence from downregulated genes by DNMT3B (Fig. 1d). We are aware that these experiments were performed in stem cells and reflect the methylation landscape set up by DNMT3B in cells where it has high levels of expression relative to differentiated cells. In the context of cancer, it has been reported that embryonic or undifferentiated cells have similarities to cancer cells^93^. Although they should be taken with caution, the coincidences found to reflect strong similarities between what was found in HaCaT cells with DNMT3B overexpression and what was reported in stem cell models. The P-down-3B genes were found involved in several key signaling pathways downregulated in cancer (Fig. 1b,c), and some of them impact processes such as cell migration. We found that the DNMT3B overexpression increased the migration in HaCaT cells (Fig. 6). The role of DNMT3B in this process was also observed in bladder cancer upon DNMT3B knockdown inhibited migration and invasion^48,94^. Our results suggest that the DNMT3B overexpression is enough to downregulate several genes. These genes are involved in the process closely related cancer and have *cis* elements associated with gain de novo methylation in promoters.

Cancer cell lines are good study models for investigation of the heterogenous factors that contributed to a specific cancer type, nevertheless, with immortalized keratinocytes model (HaCaT) we trying to emulate the onset stage and a first-line event in cancer, DNMT3B up-regulation. This model may explain aberrant CGI methylation in promoters. Our approach was downregulated genes with CGIs but is important to announce that DNMT3B overexpression also was related to the up-regulation of several genes. The methylation in intragenic CGIs^51^ is related to cell differentiation^52,95^, active transcription, and enrichment of histone modifications such as H3K36me3^50^. We studied genes possibly related to the catalytic DNMT3B function, and a limitation of our study is that we can’t discriminate whether the change observed in all the deregulated genes is a catalytic or accessory function of DNMT3B^96^, we specifically tested the catalytic function in the 4 selected genes.

The line of evidence suggests that DNMTB overexpression leads to gene deregulation, which occurs in more than one type of cancer. An exhaustive exploration of those genes indistinctly methylated in more than one type of cancer is necessary. The *cis* elements can be studied further as possible predictors of abnormal gene methylation.

## Conclusion

In this study, we describe some *cis* elements that were found in downregulated genes by DNMT3B overexpression. The most common element in these genes was the CGI, followed by the common motifs and TFBS. The presence of all or some of these elements could suggest a predisposition of certain regions to gain methylation in abnormal contexts. Genes with *cis* elements in this work are decreased and gain methylation by DNMT3B overexpression. The global role of DNMT3B impacts key biological processes such as cell migration.

## Methods

### Cell culture

The HaCaT immortalized human keratinocyte cell line was purchased from the American Type Culture Collection (ATCC, USA). HaCaT were grown in DMEM/F12 1: 1 medium (Sigma-Aldrich) supplemented with 10% fetal bovine serum (FBS, PAA Laboratories GmbH, Austria), 100 U/mL of penicillin (Penprocilin 800,000 U, Lakeside, Mexico) and 100 µg/mL of streptomycin (Sulfestrep, Pisa Laboratories, Mexico). The cells were grown at 37 °C in presence of 5% CO2.

### Expression systems

We use the construction pCDNA3.1-DNMT3B generated by Peralta-Arrieta et al*.*, 2017a). ~ 25 × 10^3^ HaCaT cells were transfected with 3.5 μg of pcDNA3.1-DNMT3B or 3.5 μg de pCDNA3.1(+) using lipofectamine 2000 Reagent (Lipofectamine, 2000 *Invitrogen, Life Technologies, Carlsbad, CA, USA*). After 48 h pos-transfección, the HaCaT cells were harvested for RNA *(Direct-Zol RNA miniprep, ZymoResearch)* and DNA (*Wizard® Genomic DNA Purification, Promega Madison, USA)* extraction.

The HaCaT with stable expression of DNMT3B was generated by the dCAS9 system. For the design and evaluation of guide sequences for the CRISPR-Cas9 system, we used the CRISPOR software (http://crispor.tefor.net/). The human sequence of DNMT3B promoter was a user for guide design. Selected guide sequences had among the lowest off-target scores. Sequences for all guides used in this study are provided in Supplementary File 1, Supplementary Table 1. Oligonucleotides for guides were cloned into lentiSAM-v2 (Puro) (Addgene Plasmid #92,062). Plasmids were expanded and purified using a Qiagen MiniPrep kit prior to lentiviral transfection. We performed the transfection in HaCaT cells with sg-1/sg-2 (closer to TSS) and with sg-3/sg-4. Transfected cells were placed at 37 °C and 72 h after transfection cells were re-suspended in fresh medium supplemented with 5 μg/mL of Puromycin (Sigma) and transferred into a well of a 6-well plate. After 6 days (9 days post-transfection) an aliquot of cells was used for RNA extraction and the gene expression was quantified for RT-PCR.

### Bioinformatic analysis: identification of CpG islands, Gene Ontology, consensus sequences of methylated promoters, and transcription factors binding sites

Previously, our laboratory group analyzed global expression genes (H35K microarray) in HaCaT cells with DNMT3B overexpression at 48 h^37^. We selected a list of 252 genes that decreased their expression with a z score of −2.0 to −6.8 in the H35K microarray. In these genes, we selected the regulatory region covering from −2000 to + 1000 bp relative to the transcription start site. The sequences of the genes were downloaded from the Eukaryotic promoter Database (https://epd.epfl.ch//index.php). CpG islands prediction was carried out with the MethPrimer program (https://www.urogene.org/cgi-bin/methprimer/methprimer.cgi). CGI). The criteria for considering a CpG island were a region > 300 bp, a percentage of GC above 50%, and an expected/observed ratio ≥ 0.6 analyzed in dbCGI (CpG island annotation tool). The consensus sequences analysis was made in downregulated genes with CpG islands in MEME Suite (Multiple Em for Motif Elicitation) (http://meme-suite.org/). All TFBS on the regulatory region of P-down-CGI were found with Alibaba 2.0 (http://gene-regulation.com/pub/programs/alibaba2/) and we overlap dates with transcription factors with experimental evidence of direct interaction with DNMT3B. We take genome browser (https://genome.ucsc.edu) information for the enrichment of transcription factors on the P-down-3B. GO analysis was performed with the Gene Functional Annotation Tool at the DAVID website (https://david.ncifcrf.gov/, version 6.8). Default parameters were used for the enrichment analysis for BP, cellular component (CC), and molecular function (MF). The resulting GO terms and the corresponding p-values were then processed using REVIGO to remove redundancy. The ten most significant BP categories were shown. Data mining. Data obtained from the Gene Expression Profiling Interactive Analysis (GEPIA; http://gepia.cancer-pku.cn/) database was utilized to analyze the mRNA expression level of DNMT3B in six cancer and normal cervical tissues. The association between a high and low DNMT3B mRNA expression with the overall survival (OS) of patients with cancer was analyzed by Kaplan‑Meier analysis, and the hazard ratio (HR) and log‑rank P‑value were also computed.

### RNA extraction and Quantitative-RT-PCR

Total RNA was extracted from HaCaT cells transfected by 48 h with pCDNA3.1-DNMT3B or pCDNA3.1( +) by Trizol *(Direct-Zol RNA miniprep, ZymoResearch)*. 100 ng total RNA was used for RT-qPCR with the *kit KAPPA TM SYBR®FAST One-Step (Kapa Biosystems, Boston, Massachusetts, USA).* Primer sequences are shown in (Supplementary File 1, Supplementary Table 1).

### Methylation specific PCR (MSP)

For MSP, 2 μg of DNA of HaCaT cells transfected with con pCDNA3.1-DNMT3B or pCDNA3.1( +) by 48 h were treated with an *EZ DNA Methylation-Gold™* kit (*ZymoResearch, Irvine CA the USA*). MSP primers are shown in Supplementary File 1, Supplementary Table 1 and were performed in a total of 10 µL, containing 1 µL of bisulfite-treated DNA, 250 nM of each primer, and AmpliTaq Gold360 Master Mix (Applied Biosystems, *Foster City, California, USA*).

### Wound healing assays

Control HaCaT cells and HaCaT cells with DNMT3B overexpression were grown until confluence on 60-mm culture dishes supplemented with DMEM/F12 as described earlier. Cells were starved for 24 h in DMEM/F12 without FBS and treated for 2 h with Cytosine β-D-Arabinofuranoside (AraC) to inhibit cell proliferation during the experiment. After starvation, cells were scratch-wounded using a sterile 200 μL pipette tip, suspended cells were removed by washing with PBS twice, and the cultures were re-fed with DMEM/F12. The progress of cell migration into the wound was monitored at 0 and 24 h using an Olympus BX43 microscope with a 10 × objective. The bottom of the plate was marked for reference, and the same field of the monolayers was photographed immediately after performing the wound (time = 0 h), and 24 h after treatments, five images per plate were analyzed. The distance between the edges of the wound was measured at times 0 and 24 h, and the reported migrated distance corresponds to the difference between these two. The migration area was determined by measuring the total area of the wound using the Image J software and the MRI wound-healing tool.

## Supplementary Information

Supplementary Information 1.

Supplementary Information 2.

## Data Availability

All data analyzed during this study are included in this published article and its supplementary information files^37^. The datasets used in figures supplementary 4 and 5 were downloaded from Gene Expression Omnibus (GEO) database under accession numbers GSE65838 and GSE150072, respectively.
