# Supplementary material for: DNMT3B overexpression downregulates genes with CpG islands, common motifs, and transcription factor binding sites that interact with DNMT3B
Source: Sci Rep. 2022 Dec 2;12:20839. doi: 10.1038/s41598-022-24186-6 (PMC9718745; doi:10.1038/s41598-022-24186-6)
Supplement: Supplementary file 1 — Supplementary Information 1. [file 41598_2022_24186_MOESM1_ESM.docx]

**
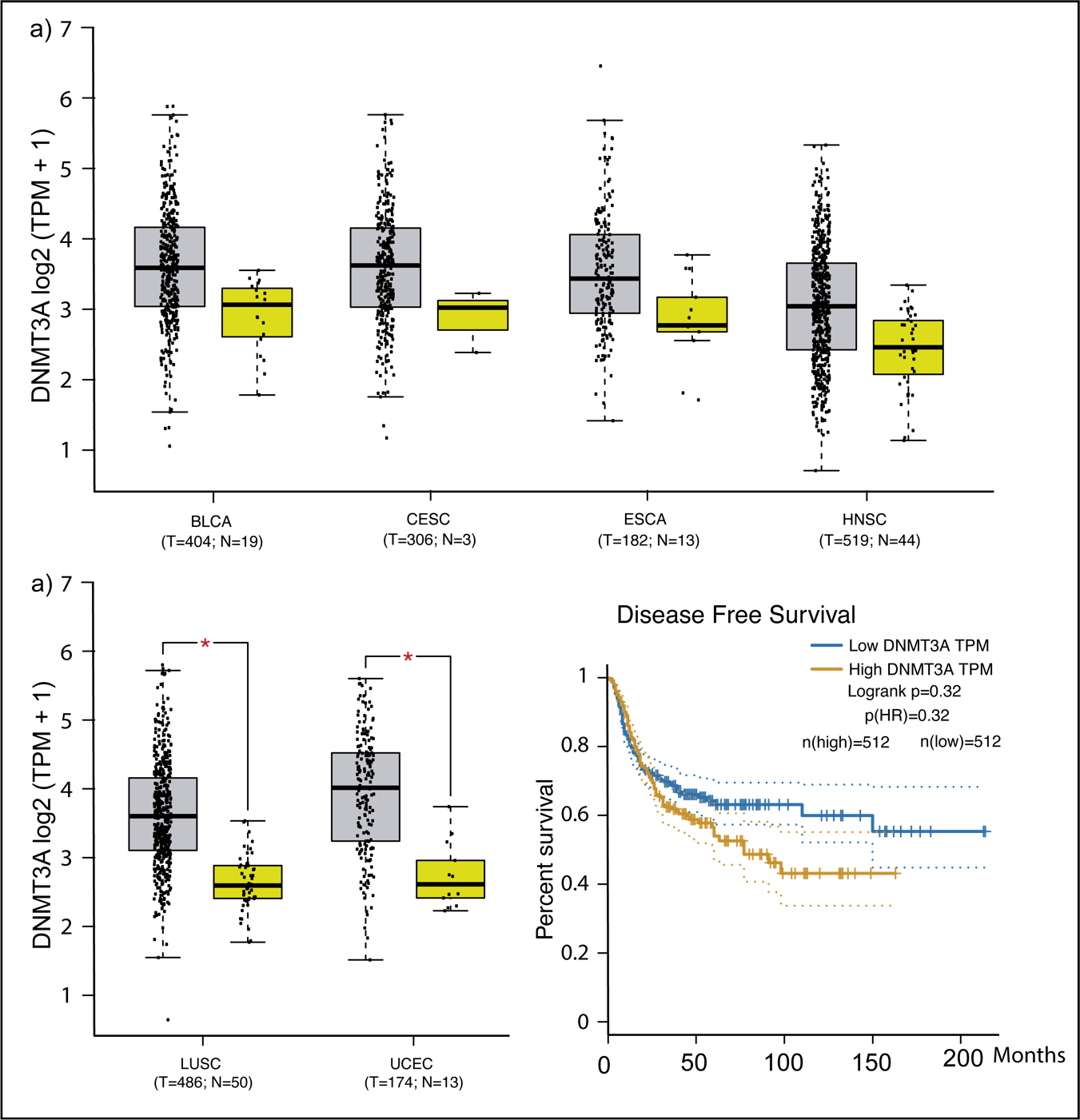
**

**Supplementary Figure 1** DNMT3A expression in patients’ tumors from TCGA data sets and correlation with prognosis. a) Comparison of the DNMT3A expression in six carcinomas with respect to normal tissue using data retrieved from TCGA. b) Kaplan-Meier curves of disease-free survival for six solid tumors (a) with low versus high expression of DNMT3A. BLCA, Bladder Urothelial Carcinoma; CESC, Cervical Squamous Cell Carcinoma and Endocervical Adenocarcinoma; ESCA, Esophageal Carcinoma; HNSC, Head and Neck Squamous Carcinoma; LUSC, Lung Squamous Cell Carcinoma; UCEC, Uterine Corpus Endometrial Carcinoma; T, Tumor; N, Normal tissue.

**
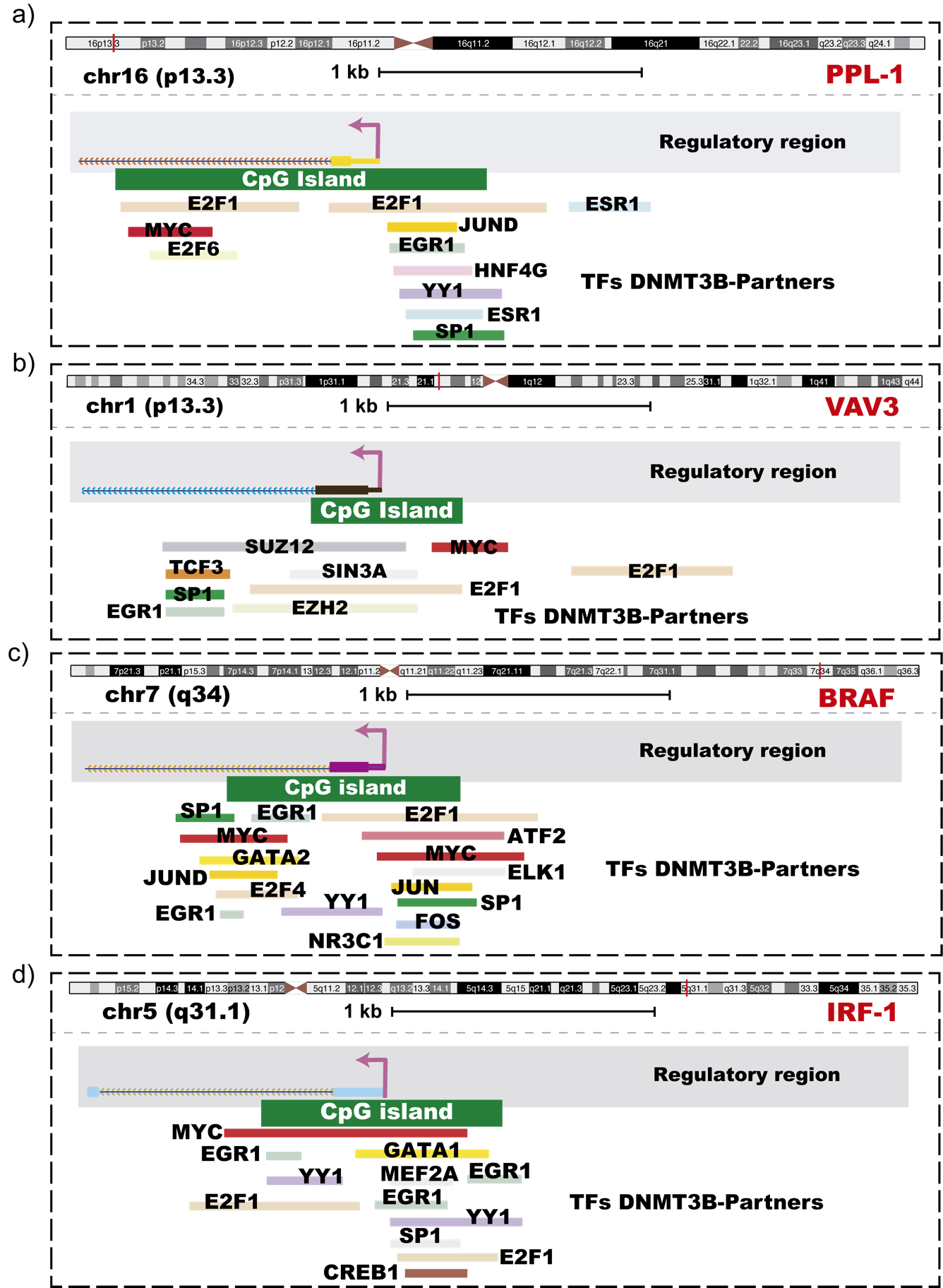
**

**Supplementary Figure 2** Cis elements found in genes downregulated by DNMT3B. a) PPL1, b) VAV3, c) BRAF and, d) IRF1 genes.

**
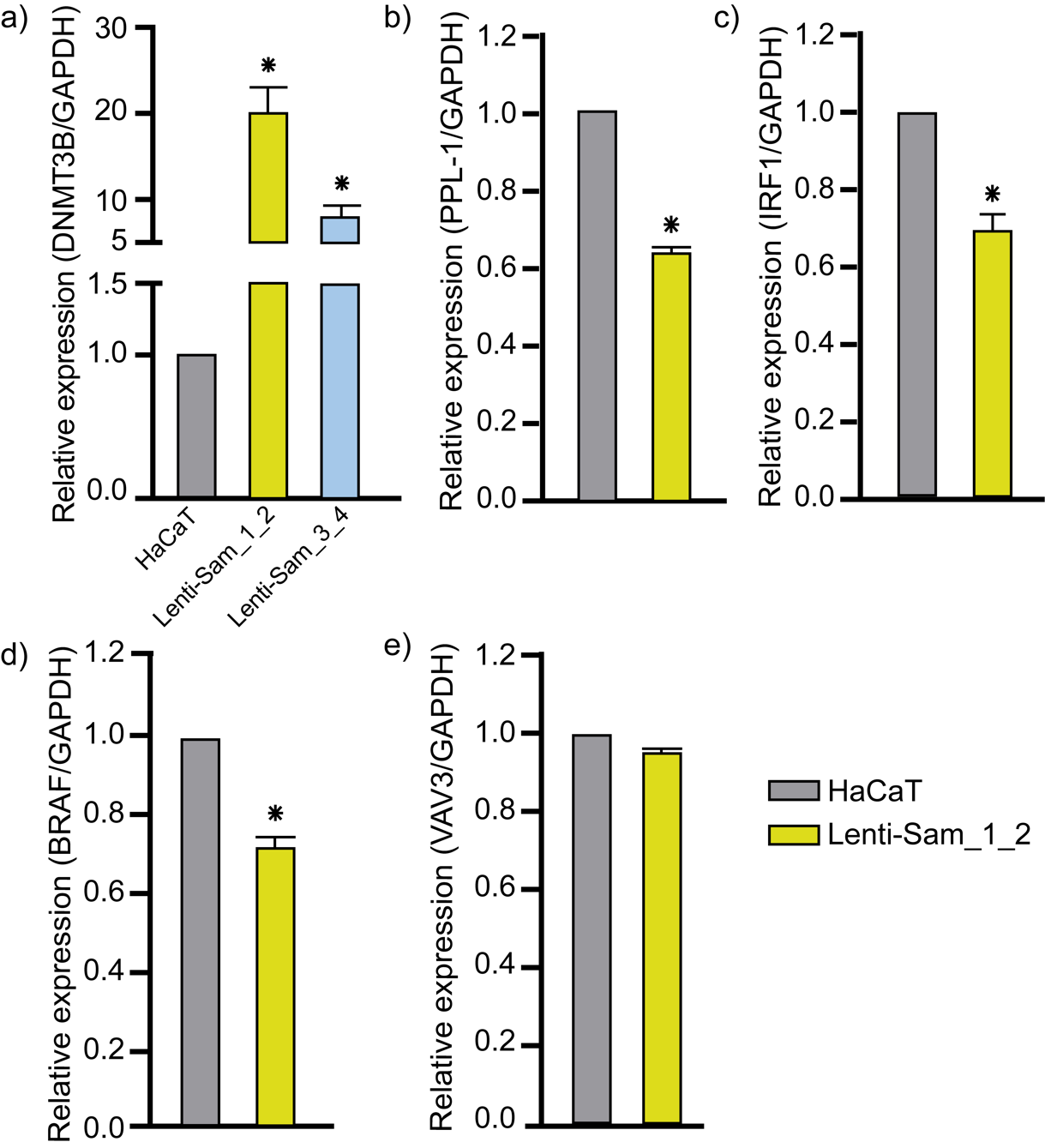
**

**Supplementary Figure 3** Endogenous overexpression of DNMT3B results in decreased expression of target genes. a) mRNA expression of DNMT3B in HaCaT with two sg-RNA combinations. b), c), d), and e) mRNA expression of selected genes in HaCaT cells and HaCaT cells with DNMT3B overexpression (Lenti-Sam_1_2). *p>0.05 of three biological replicas by triplicate.

**Supplementary Figure 4** RNA-Seq data from Human Epidermal Stem Cells (EpSC) with DNMT3B depletion. PPL1 and IRF1 increase their expression in EpSC with si-DNMT3B.


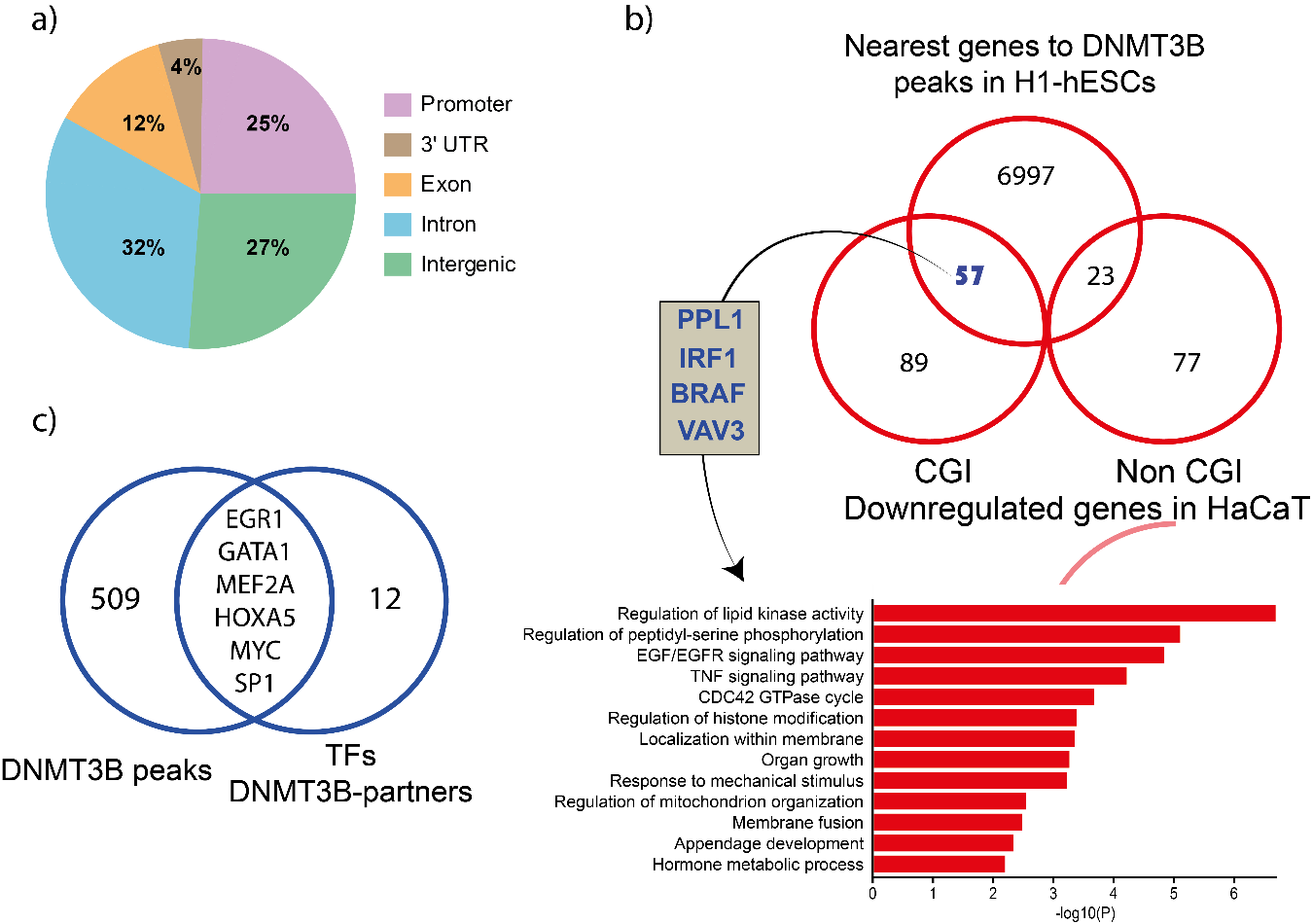


**Supplementary Figure 5** DNMT3B ChIP-sequencing in H1-hESC. a) Genomic distribution of DNMT3B peaks. b) Nearest genes to DNMT3B peaks are found in H1-hESC and overlap with downregulated genes in HaCaT cells with and without CpG island. GO of 57 genes with CpG island, downregulated and with DNMT3B peaks (are denoted the genes four of interest). c) Overlap between DNMT3B peaks of transcription factors in H1-hESCs and binding sites for transcription factors in downregulated genes in HaCaT Cells.


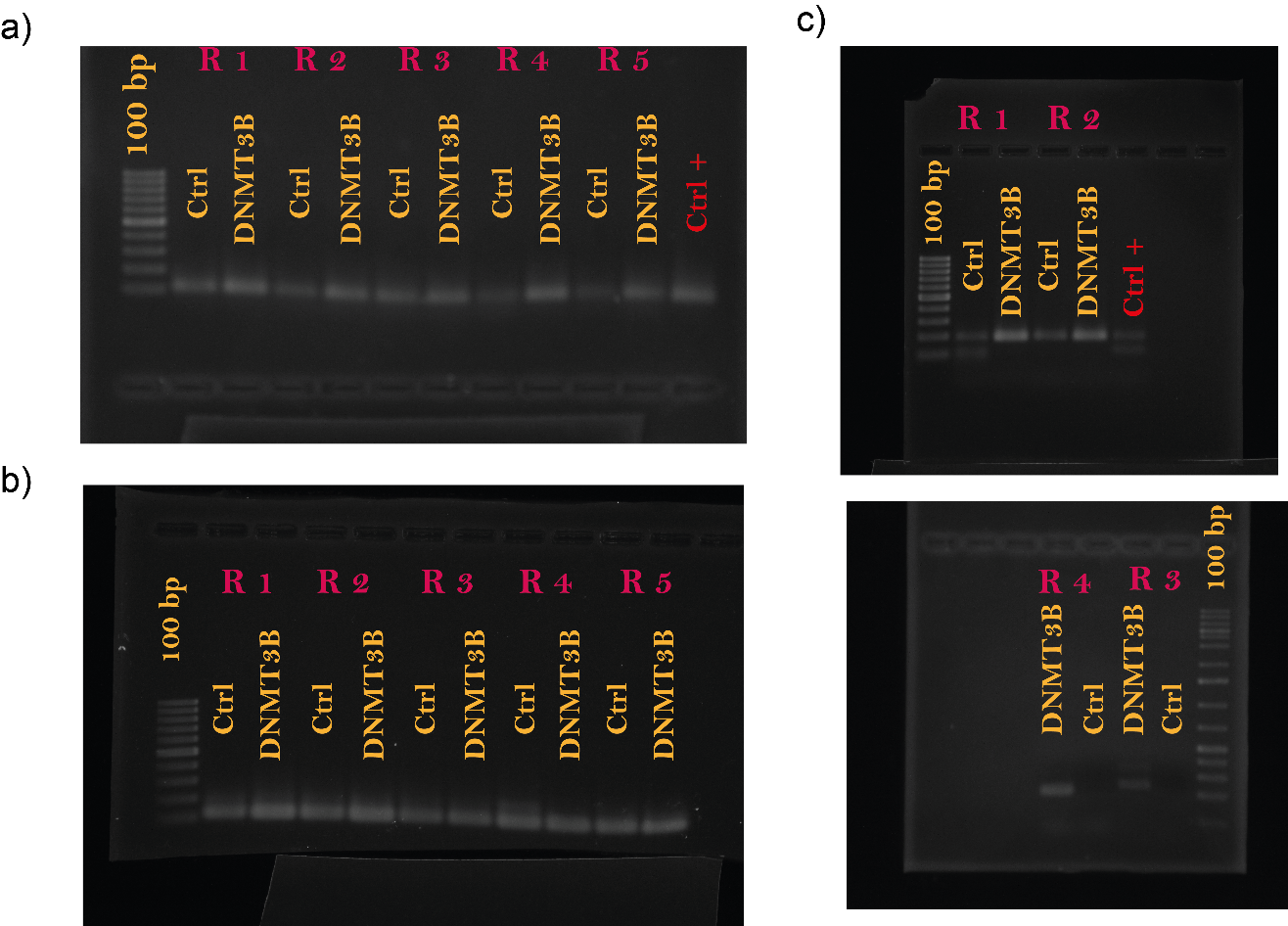


**Supplementary Figure 6** Original photographs from agarose gels of analyzed genes. The PCR-SM products were loaded and resolved in agarose 1.5% gels; a) IRF1, b) BRAF, and c) PPL1.
