# Supplementary material for: DNMT3B overexpression downregulates genes with CpG islands, common motifs, and transcription factor binding sites that interact with DNMT3B
Source: Sci Rep. 2022 Dec 2;12:20839. doi: 10.1038/s41598-022-24186-6 (PMC9718745; doi:10.1038/s41598-022-24186-6)
Supplement: Supplementary file 2 — Supplementary Information 2. [file 41598_2022_24186_MOESM2_ESM.docx]

| **Supplementary Table 1**. Primer sequencies | | | |
| --- | --- | --- | --- |
| Gene | Forward | Reverse | Tm °C |
| RT-qPCR |  |  |  |
| GAPDH | CCGGGAAACTGTGGCGTGATGG | AGGTGGAGGAGTGGGTGTCGCTGTT | 60 |
| DNMT3B | ACCACCTGCTGAATTACTCACG | GATGGCATCCATCATCACTGG | 60 |
| PPL | CGGAGCATCTCTAACAAGGA | CACGATGTTCTTCTCCACCT | 62 |
| IRF-1 | GCATGAGACCCTGGCTAGAGAT | TTTCCCCTGCTTTGTATCGG | 62 |
| BRAF | GAGCATAATCCACCATCAAT | CTGTTGTTCTCTTTGTTGGA | 60 |
| Name SgRNA | Sequence 5´- 3´ | |  |
| DNMT3B-sg1 | GAGGGAAATTTGAAATCGCT | |  |
| DNMT3B-sg2 | GATTTCACTGGCCCGGGGTT | |  |
| DNMT3B-sg3 | CGGGCTCCTTTCAACCCGAA | |  |
| DNMT3B-sg4 | AGTGGTTCAATGGTCATCCC | |  |
